# Functional extra-adrenal paraganglioma of the retroperitoneum giving thoracolumbar spine metastases after a five-year disease-free follow-up: a rare malignant condition with challenging management

**DOI:** 10.11604/pamj.2017.28.94.13783

**Published:** 2017-09-29

**Authors:** Stylianos Kapetanakis, Danai Chourmouzi, Grigorios Gkasdaris, Vasileios Katsaridis, Eleftherios Eleftheriadis, Panagiotis Givissis

**Affiliations:** 1 Spine Department and Deformities, Interbalkan European Medical Center, Thessaloniki, Greece; 2 Radiology Department, Interbalkan European Medical Center, Thessaloniki, Greece; 3 Neurosurgery Department, Interbalkan European Medical Center, Thessaloniki, Greece; 4 Pathology Department, Interbalkan European Medical Center, Thessaloniki, Greece; 5 First Orthopaedic Department of 'Aristotle University of Thessaloniki', 'Papanikolaou' Hospital, Exohi, Thessaloniki, Greece

**Keywords:** Paraganglioma, retroperitoneum, metastasis, vertebral

## Abstract

Paragangliomas are benign neoplasms that arise from the autonomic nervous system and the associated paraganglia. Although benign, they have been shown to possess metastatic potential. Extra-adrenal retroperitoneal paraganglioma with vertebral metastasis is considered very uncommon. Here, we present a case of a functional extra-adrenal paraganglioma of the retroperitoneum giving metastasis to T4 vertebra after five years of follow-up in a 48-year-old man who had been initially treated with complete resection of the primary tumor. The condition of the patient improved significantly after radiosurgery and somatostatin analogs treatment, until lumbar spine lesions appeared six months later. Our case demonstrates that retroperitoneal paraganglioma is a rare condition which should be considered in the differential diagnosis of a retroperitoneal mass combined with vertebral lesions. Additionally, increased physician awareness and long-term follow-up is mandatory for all patients with history of retroperitoneal paraganglioma since metastases may occur after long latent intervals from the initial diagnosis.

## Introduction

Paragangliomas, also known as extra-adrenal pheochromocytomas, are neuroendocrine tumors that arise from chromaffin cells of the adrenal medulla and from neuroendocrine cells of the extra-adrenal autonomic paraganglia, with the majority of the tumours being benign in nature [1]. There have been several reports of metastatic paraganglioma in the literature, but rarely do such tumors metastasize to the spine [2, 3]. In this case report, the patient was diagnosed with a metastatic lesion in T4 vertebra after five disease-free years from the initial resection of a retroperitoneal paraganglioma.

## Patient and observation

**Figure 1:**
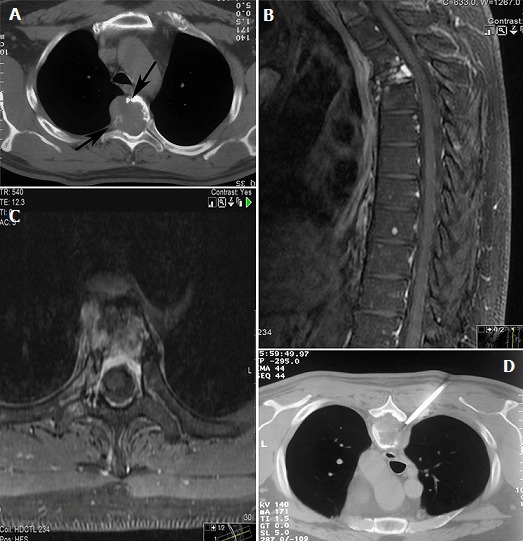
(A) CT scan depicts an expansile lytic lesion in T6 vertebra that destroys the cortex of the vertebral body (arrows), Sagittal; (B) and axial; (C) fat-saturated (FS) T1-weighted MRI with intravenous gadolinium contrast demonstrates compression fracture of vertebral body and the extent of disease in the marrow cavity, soft tissues and epidural space; (D) computed tomography guided needle biopsy

**Figure 2:**
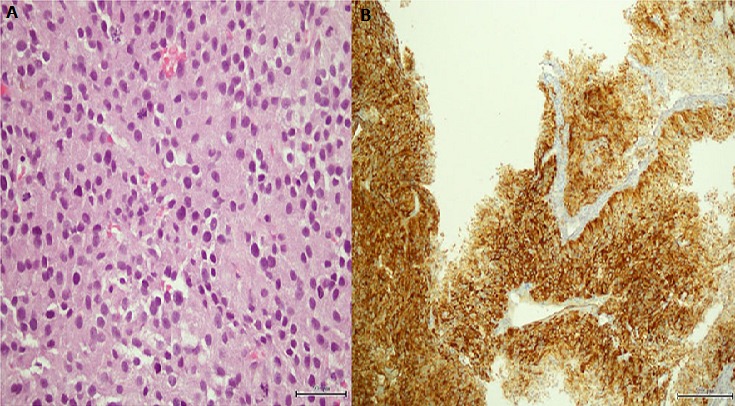
(A) tumor cells arranged in solid nests with increased number of mitoses; (B) tumor cells positive for chromogranin A

**Figure 3:**
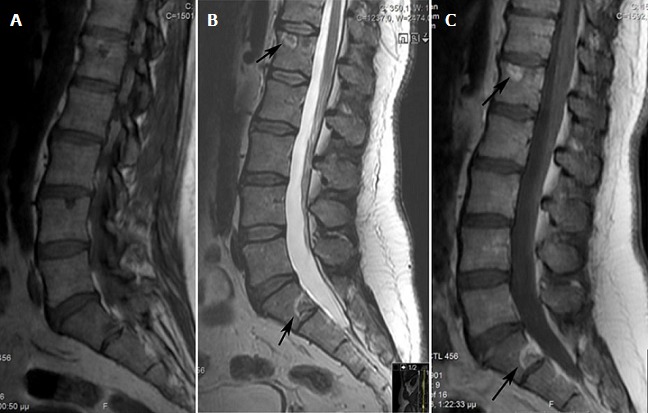
MRI of the lumbar spine, sagittal section, T1: (A) T2; (B) and gadolinium-enhanced T1-weighted; (C) sequences: presence of nodular bone metastases with a halo (arrows)

A 48-year-old man was admitted with the complaints of worsening back pain, palpitation, headache and sweating. He denied any trauma or weakness in his extremities. On physical examination he had point tenderness in his thoracic vertebrae and blood pressure 185/84 mm Hg. The patient had a history of a retroperitoneal paraganglioma that was discovered by chance at the age of 43 as an abdominal mass. No family history of paraganglioma was detected. The lesion was completely resected through laparotomy and the patient was found to be disease-free at follow-ups. When admitted to our hospital, laboratory tests revealed elevated serum normetanephrine and norepinephrine. CT scan revealed a lytic lesion involving the T4 vertebral body (Figure 1 A). On MRI, a compression fracture of the vertebral body with small epidural component was depicted (Figure 1 B,C). The supposed spinal metastatic functional paraganglioma was confirmed by computed tomography guided needle biopsy (Figure 1 D). The histologic and immunohistochemical assays revealed a paraganglioma (Figure 2 A,B). The patient displayed significant improvement in pain after radiosurgical treatment. Also, the patient underwent somatostatin analogs therapy and showed symptomatic and biochemical improvement as well. However, MRI revealed small new lesions in lumbar spine 6 months later (Figure 3 A,B,C). Treatment with combination chemotherapy for the management of the disease is now under discussion by a multidisciplinary team.

## Discussion

Most commonly, paragangliomas are seen within the adrenal medulla giving rise to pheochromocytoma and only 10% of paragangliomas arise outside the adrenal glands. They can be functional with symptomatology of paroxysmal hypertension, palpitation, headache, sweating or be detected as non-functional, asymptomatic and slow-growing [4]. It is hard to make a correct initial diagnosis. CT, MRI or ultrasonography are capable of detecting a retroperitoneal mass, however diagnosis of the retroperitoneal mass still relies on the postoperative histopathological results, since benign and malignant paragangliomas may exhibit similar clinical symptoms and imaging findings. Apart from central necrosis, vascular and lymphatic invasion and mitotic abnormalities; chromogranin A, vimentin, S-100, synaptophysin can be used in the immunohistochemical analysis of paragangliomas and indicate the true nature of the tumour [3]. In our case, vascular invasion and increased number of mitoses were observed and also the tissue was positive for chromogranin A. Malignant paraganglioma is a rare presentation diagnosed by local recurrence after total resection of the primary mass or findings of distant metastasis [3, 5]. The time intervals for recurrence may be from months to many years. In our report, the patient presented a metastatic lesion five years after the initial resection. Several authors report that vertebral metastases from malignant paragangliomas of the retroperitoneum are rare and late in their emergence as well. Kitagawa et al reported a case of T6 metastasis from retroperitoneal paraganglioma 9 years after the primary surgery [6]. Doza et al reported local recurrence and vertebral metastases 10 years after first surgical resection [7]. Awareness of imaging manifestations of spinal metastatic disease is essential. We consider that in cases like this, MRI is the most useful imaging technique; because contrary to x-rays, bone scintigraphy and CT scans, it allows both direct visualization of bone marrow involvement with high spatial resolution, as well as, early diagnosis of possible neural compression [8]. In comparison to a similar case management by He et al our patient did not have any SPECT (single photon emission computed tomography) imaging [3].

In order to reach a histological confirmation of the metastatic lesion, core needle biopsy under CT guidance was performed. We decided to the use of this technique as a confirmation tool, because it is considered a simple, minimally invasive method, with high levels of diagnostic accuracy in cases of suspected spinal metastatic lesion [9]. In the literature, two types of lesions have been described. Expanding lesions are characterized by cortical destruction with bone marrow and soft tissue involvement, while nodular lesions present a specific radiological appearance, consisting of a central low-intensity signal surrounded by a single fat-like halo or a double halo with a fat-density inner circle and an outer circle suggestive of edema. In some patients, these lesions can coexist [10]. The patient was diagnosed with an expanding lesion on T4 vertebral body and six months later with nodular lesions involving lumbar vertebrae. In our opinion, expanding form of metastasis is related to evolution from the nodular form and coexistence is seen in the progressive disease. As regards to treatment, while the individual behavior of a paraganglioma is unpredictable, the primary therapeutic option should be directed towards the complete surgical resection of the primary tumor with meticulous preservation of vital neurovascular structures [11]. Our case was treated in a similar way concerning the primary site. When metastatic, it is believed that vertebral metastases with neural compression should be treated with decompressive surgery and total en bloc spondylectomy. He et al treated a similar case of metastatic retroperitoneal paraganglioma with resection of the vertebral metastasis and posterior fixation surgery. To avoid serious complications associated with surgical resection, our team concluded that the best appropriate initial case management would be radiosurgery and somatostatin analogs treatment. Although the ideal final treatment remains unclear, concomitant treatment with radiosurgery and reconstructive surgery has appeared to be both safe and effective [12]. Somatostatin analogs have been an effective complementary treatment for metastatic paragangliomas as well [13]. Hruby et al stated that external beam radiotherapy, chemotherapy and 131I metaiodobenzylguanidine can be effective treatment options [14]. Further studies should examine the efficacy and safety of the existing therapeutic approaches regarding the metastatic to the spine retroperitoneal paraganglioma.

## Conclusion

In conclusion, we report a case of a functional extra-adrenal paraganglioma of the retroperitoneum giving vertebral metastases five years after the complete removal of the primary site. Malignant paraganglioma with spinal metastases is a rare presentation and metastases' appearance can vary from months to many years after the initial diagnosis. Imaging modalities have crucial role in the diagnosis and evaluation of spinal metastatic disease. Treatment should be individualized according to each patient and surveillance with clinical assessment, imaging tests and long-term follow-up is mandatory in patients with history of retroperitoneal paraganglioma. Finally, the combination of aggressive behaviour and delayed reappearance is something that we should keep in mind; and certainly this case represents a good example.

## Competing interests

The authors declare no competing interests.
